# Azygos Vein Pseudoaneurysm After Trauma: A Case Report

**DOI:** 10.7759/cureus.51553

**Published:** 2024-01-02

**Authors:** Kyle Nugent, Andrew McCague, Sage Rivard, Austin Henken-Siefken

**Affiliations:** 1 Surgery, Desert Regional Medical Center, Palm Springs, USA; 2 Trauma and Acute Care Surgery, Desert Regional Medical Center, Palm Springs, USA

**Keywords:** pseudoaneurysm, azygos vein, trauma, motor vehicle accident, azygos vein pseudoaneurysm

## Abstract

High-speed motor vehicle collisions (MVCs) often result in severe musculoskeletal, neurological, and vascular injuries. Among these, azygos vein pseudoaneurysms (AVPs) are a rare and potentially life-threatening vascular complication. Our case study highlights an instance of an AVP arising from a high-velocity MVC, underscoring their critical significance in trauma scenarios. Additionally, this report delves into the complexities of managing AVPs, both traumatic and idiopathic, emphasizing the urgent need for intervention and the intricacies of their treatment.

## Introduction

Azygos vein pseudoaneurysms (AVPs) are infrequent but potentially fatal vascular complications that can occur after high-speed motor vehicle collisions (MVCs). These conditions impact the azygos vein, a crucial conduit responsible for transporting blood from the back of the chest to the heart. While AVPs can result from various causes, including trauma, invasive medical procedures, or idiopathic factors, their occurrence following MVCs is relatively rare. The azygos vein, situated in the posterior mediastinum, plays a pivotal role in maintaining proper blood circulation within the chest cavity. However, the high-energy impact associated with MVCs can lead to trauma to this delicate vascular structure. When the azygos vein's wall experiences tearing or rupture due to the force of the collision, it can result in the accumulation of blood outside the vessel, leading to the formation of an AVP. The consequences of AVPs following MVCs can be severe and complex. These pseudoaneurysms can vary in size and may rupture, causing significant bleeding and hemodynamic instability. Therefore, a prompt diagnosis and intervention are crucial in effectively managing such cases.

## Case presentation

**Figure 1:**
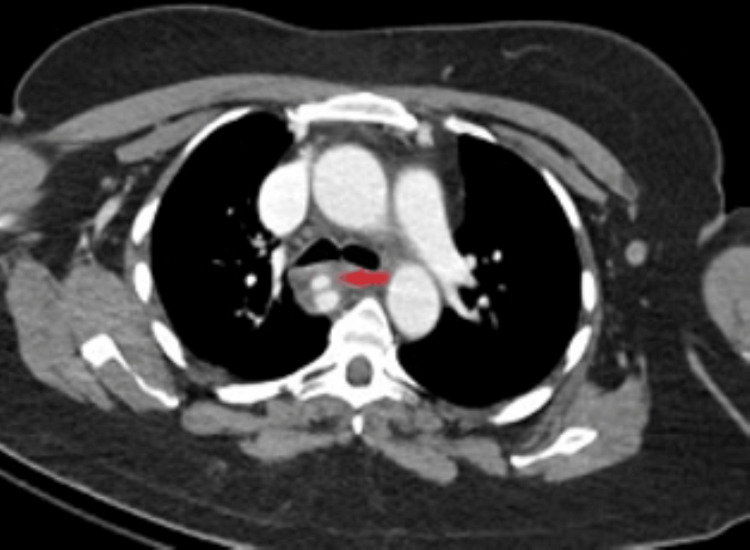
CT with contrast showing an azygos vein injury and pseudoaneurysm. Red arrow points to the area of injury

A 55-year-old female presented to the emergency room (ER) after suffering an AVP following a high-speed MVC. She was the driver of a car that was struck on the passenger side at approximately 55 miles per hour, resulting in extensive vehicle damage and 12 inches of intrusion into the passenger space. Despite wearing a seatbelt and the airbags deploying, she sustained multiple injuries. Upon arrival at our level I trauma center, the patient underwent primary and secondary surveys. Her complaints included right chest wall and breast pain, upper back discomfort, and right flank pain. An Extended Focused Assessment with Sonography in Trauma (eFAST) exam was positive for lack of lung sliding at her right lung. Medical assessment revealed stable vital signs, including a blood pressure reading of 155/88 mmHg, a heart rate of 90 beats per minute, a respiratory rate of 22 breaths per minute, and an oxygen saturation level of 97% while receiving 4 liters of oxygen. Additionally, the Glasgow Coma Scale (GCS) score was recorded as 12. Admitting laboratory values are presented in Table 1. The patient denied any past medical history or surgeries and denied any allergies or medication use. She was taken for a whole-body CT and found to have significant injuries including a right AVP, right 10-11 rib and right L1-L2 transverse process lumbar fractures, a small right pneumothorax, a right breast contusion, a right retroperitoneal paravertebral hematoma, a grade 1 splenic laceration, a grade 4 right renal laceration, a faint abdominal wall seatbelt sign, and a small hemoperitoneum (Figure 1). 

**Table 1:** Admitting laboratory values

Test	Results	Reference range
White blood cells	21.7	4-10x10^3^/μL
Hemoglobin	12.6	12-17 g/dL
Hematocrit	39.2	40-51%
Platelet	318	150-400x10^3^/μL
Sodium	139	136-146 mmol/L
Potassium	4	3.5-5.1 mmol/L
Chloride	107	98-106 mmol/L
Carbon dioxide	23	23-29 mEq/L
Glucose	183	80-110 mg/dL
Blood urea nitrogen	19	7-20 mg/dL
Creatinine	0.6	72-127 μmol/L

The patient remained stable throughout her primary survey and emergency department stay. Interventional radiology (IR) and cardiothoracic surgery were consulted for her injuries. Her kidney and splenic lacerations were managed nonoperatively. A repeat CT angiography with venous phase was performed to better characterize her azygos vein injury. The area of azygos vein injury was stable without sign of bleeding. A saccular pseudoaneurysm/varix was seen on imaging. Nonoperative management was planned for this injury. She was admitted to the ICU for hemodynamic monitoring. Both cardiothoracic surgery and IR recommended nonoperative management. The increased risk of bleeding from instrumentation with a wire was thought to put the patient at an increased risk for ruptures where the risk of spontaneous bleeding was felt to be low. The patient remained stable in the ICU and was transferred to the floor and then discharged home on hospital day 5. She continued to recover and was seen in the clinic two weeks later. She continued to make a full recovery.

## Discussion

**Figure 2:**
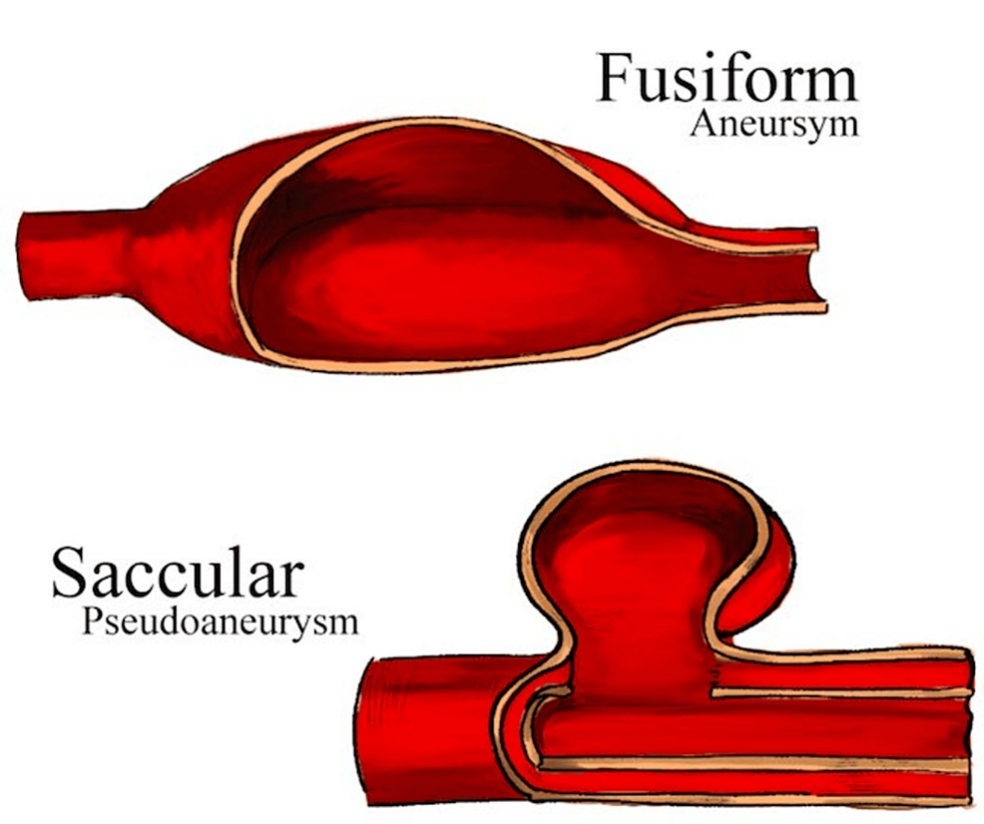
Illustration of a saccular pseudoaneurysm compared to a fusiform aneurysm Image Credit: Ms. Debbie Kwon; published with permission

The azygos vein plays a crucial role in draining blood from the back of the chest and carrying it to the heart. When a tear or rupture occurs in the azygos vein wall, blood accumulates outside the vessel, confined by nearby tissues or structures leading to an AVP. Damage to the azygos vein can result from trauma, injury, complications from invasive medical procedures, or idiopathically. There is no precise data on the exact causes of azygos vein injuries, but most AVPs are generally deemed idiopathic [1]. Yet, occurrences of AVPs resulting from traumatic incidents, such as motor vehicle accidents (MVAs), or chest injuries, such as stabbings or penetrating wounds, are relatively rare. A retrospective review of one institution's surgical department database revealed only seven cases of azygos system venous injury after trauma within an 11-year time frame [2]. In addition to their rarity in the emergent setting, AVPs have relatively high mortality rates, especially when associated with concomitant injuries. In one study, during a 40-year period, a retrospective analysis was conducted on a vascular injury database. It revealed 22 injuries within the azygos venous system: 21 in the azygos vein and one in the hemiazygos vein. These injuries resulted from penetrating trauma, comprising 19 gunshot wounds and three stab wounds. All instances were attributed to penetrating trauma, with accompanying lung injuries seen in each case. Additionally, injuries to neighboring structures such as aortic branches were noted, along with concomitant damage to major vessels such as the subclavian or splenic arteries observed in the reported cases [3]. In rare instances, traumatic pseudoaneurysms might involve cerebral arteries, like the anterior cerebral artery (ACA), due to avulsion from trauma [4]. In our patient's case, her traumatic AVP manifested as a saccular pseudoaneurysm/varix (Figure 2). The conjunction of these accompanying injuries and the potential for severe bleeding or hemodynamic instability upon pseudoaneurysm rupture significantly contribute to the lethal nature of AVPs as well as warrant its prompt intervention. 

Patients experiencing AVPs after trauma often exhibit various symptoms such as chest pain, respiratory distress, hemoptysis, hemorrhage, upright dyspnea with relief in the supine position, or back pain [5]. In the emergency department, individuals with these symptoms following trauma, or suspected AVPs, should undergo assessments such as CT angiography or MRI scans to diagnose and assess potential vascular injuries [6]. However, due to the rarity of traumatic AVPs seen in emergency departments, no standardized emergency protocol exists for traumatic AVPs. Instead, the accepted approach involves swift assessment for symptom severity and potential complications like bleeding or tissue compression. Specialists, such as vascular surgeons or interventional radiologists, are consulted for evaluation and possible intervention. Imaging, notably CT or MRI scans, helps gauge the pseudoaneurysm's size, location, and potential complications. The management of AVPs following MVAs have evolved, with traditional approaches involving open surgeries now being supplemented by endovascular treatments. This approach involves using catheters and stents to address the injury, providing a less invasive alternative to open surgery [7]. In general pseudoaneurysm management, flow-diverting strategies, like stent placement, have shown promise in treating similar vascular conditions [8]. However, some cases allow for conservative approaches, especially if the patient is stable. Successful conservative management, including close observation and supported care, have been reported [9]. Regardless of the chosen treatment, prompt intervention is crucial to prevent potential complications, including hemorrhage or hypovolemic shock [10]. In determining whether to opt for surgery or conservative treatment for AVPs, there's no definitive guideline. Studies and clinical practice offer diverse perspectives without a universally accepted aneurysm size dictating surgery. Cases show successful non-surgical management regardless of size [11], yet larger AVPs pose risks of complications like rupture or thrombosis, warranting surgical consideration [12]. The necessity for surgery relies on multiple factors: a patient's symptoms, clinical condition, possible complications, and the expertise of the medical team. However, urgent intervention significantly boosts patient survival. In our case, the patient received a swift evaluation by the trauma team and consulted with cardiothoracic surgery and IR. While specific studies on the significance of immediate medical intervention for traumatic AVPs are scarce, ample evidence emphasizes the life-saving benefits of prompt action in thoracic and abdominal aortic aneurysms. Timely surgical procedures or endovascular interventions notably reduce postoperative complications, decrease morbidity rates, and enhance overall patient well-being [13].

The occurrence of azygos vein injury isn't solely linked to trauma. Instead, AVPs can also occur spontaneously, without any apparent trauma. Idiopathic AVPs are primarily identified incidentally through medical imaging or unrelated examinations; these injuries are often asymptomatic and are typically spotted coincidentally in medical evaluations. Typically located where the trachea meets the upper right lobe bronchus, idiopathic AVPs commonly lack noticeable symptoms [14]. Due to their incidental discovery, the precise cause of idiopathic azygos vein aneurysms remains unclear [15]. Thus, healthcare professionals must remain attentive to potential symptoms of idiopathic azygos vein injuries. Symptoms include dysphagia, sensations of tightness or heaviness in the chest, ongoing cough, and difficulty breathing or dyspnea. And if severe enough, symptoms can manifest as traumatic AVPs, with intense thoracic pain, specifically in the posterior thorax, discomfort when upright, and respiratory distress, along with palpitations or arrhythmias [16]. The uncommonness and ambiguous symptoms of AVPs make their diagnosis challenging, emphasizing the need for quick identification to intervene promptly. Medical professionals must be vigilant regarding these symptoms, particularly after recent chest trauma occurrences. 

## Conclusions

The significance of this case report centers on highlighting a rare instance of an AVP after a MVA. It illustrates swift evaluation, specialist assessment, and suitable conservative treatment, ultimately culminating in the patient's full recovery. It underscores the necessity for ER physicians and general practitioners to recognize AVP symptoms, whether caused by trauma or occurring spontaneously, ensuring a timely diagnosis and intervention. The natural history and optimal management strategies for these injuries continue to be areas of ongoing exploration due to their rarity and diverse clinical presentations.
